# Effect of nursing guidelines on first-year nursing students’ knowledge and reported practice towards sensory impairments among the elderly

**DOI:** 10.1186/s12877-022-03524-3

**Published:** 2022-11-21

**Authors:** Nadia Abdelnasser, Saieda Abd Elhameed Abdel Aziz, Sara Mohamed Ahmed El-Gamal

**Affiliations:** 1 Faculty of Nursing, Department of Gerontological Nursing, South Valley University, Qena, 83523 Egypt; 2 Faculty of Nursing, Department of Gerontological Nursing, Assiut University, Assuit, 71515 Egypt; 3 Faculty of Nursing, Department of Community Health Nursing, Tanta University, Gharbia Governorate, Tanta, 6632110 Egypt

**Keywords:** Elderly, Knowledge, Nursing students, Reported practice, Sensory impairments

## Abstract

**Background:**

Sensory impairments are common among older adults. These impairments have consequences on activities of daily living and communication with others. Such impairments for the elderly remain a significant public health issue globally. This study aimed to assess effect of nursing guidelines on first-year nursing students' knowledge and reported practice towards sensory impairment among the elderly.

**Method:**

A Pre- and post-test research design was utilized in this study to assess first year nursing students’ knowledge and reported practice towards sensory impairments among the elderly. It was carried out at faculties of nursing affiliated with three universities with a purposive sample (*n* = 531) of the first-year nursing students. The study was conducted in four phases: Pre-intervention assessment, nursing guidelines development, nursing guidelines implementation, and post-test after one month. The pre- and post-tests were conducted online and included three parts to collect the required data about students’ socio-demographic data, students’ knowledge about the five senses and changes in these senses among the elderly, and the students’ reported practice for coping with changes in these senses among elderly. Student t-tests and an ANOVA test were used to compare means. For qualitative data, comparison was done using chi-square. Pearson correlation coefficient was used for detecting the relations between continuous variables of the study.

**Results:**

There are statistically significant differences between the studied subjects means score knowledge and reported practice about the five senses among elderly people in the pre- and the post-tests (*P* = 0.001). At pre-test the total score mean of students’ knowledge was 24.25 while at post-test became 28.16. At pre-test the total score mean of students’ reported practice was 38.40 while at post-test became 44.43. There is a relationship between students’ knowledge and their reported practice at both pre-test and post-test with *P* value = 0.001.

**Conclusion:**

The levels of the first-year nursing students’ knowledge and reported practice of the studied sample towards sensory impairment among the elderly were improved after implementation of the nursing guidelines. So, it is recommended that these nursing guidelines could be embedded within the undergraduate curriculum. Raising students’ awareness through providing lectures, and workshops on sensory impairment among elderly and how to deal with them, and train students on how to communicate with sensory impairment among the elderly.

## Introduction

One of the most frequent chronic diseases in later life is sensory impairment. Over 2.2 billion individuals suffer vision impairments or blindness [1], and 466 million people have hearing loss that is debilitating [2]. For example, sensory impairments are a major issue for the elderly in the United States with one out of every six people having vision problems, one out of every four having hearing problems, one out of every four people having lost feeling in their feet, and three out of every four people have abnormal postural balance tests. Sensory deficits worsen as people get older. When comparing people aged 70–79 years to people aged 80 years and older, vision and hearing impairments both double, and the loss of feeling in the feet doubles by 40% [3].

Egypt has an increasing number of older adults like many other countries throughout the world. On January 1, 2020, the number of Egypt's older adult was around 7 million, accounting for 7.1% of the population, and is anticipated to rise to 17.9% by 2052 [4]. With age, visual, auditory, taste, smell, and tactile perceptions all deteriorate and become less effective, although the age of onset and pace of deterioration differ widely from person to person [5]. A growing proportion of the elderly population have vision and hearing loss as the incidence and prevalence of these sensory impairments rise with age [6]. These impairments can have consequences for everyday living. Vision impairment, for example, is significantly linked to problems with daily tasks such as walking, getting outside, and transferring in and out of a bed or a chair. Vision impairment can exacerbate falling, social isolation, and increase early admission to nursing homes [7, 8].

Age is not the only factor that contributes to a loss of sensation. Environment and diseases have a role as well, for example, loud and prolonged noise has effects on hearing, smoking reduces the taste and smell sensitivity, and diabetes mellitus influences vision. The way we see, hear, taste, smell, touch and respond to pain can all be affected by changes in sensation. Any changes have an impact on how we perceive the world and react to events [9]. A significant sensory change can deprive people of many simple pleasures while also making routine daily activities more challenging. Sensory changes might result in decreased mobility, increased dependence on others, a lack of environmental awareness, inadequate communication and interpersonal abilities, and a loss of self-esteem [10, 11]. For instance, because ‘deafness’ involves ‘social experiences’, it is likely the most significant sensory disorder. In contrast to impaired vision, deafness rarely induces empathy or comprehension [12]. For older adults with impaired vision recognizing food is typically difficult. They may not find food appealing. Some older adults’ diets are further harmed by poor health, low physical strength, the need to cook meals for several people, and limited resources [13].

Tactile feelings enable people to recognize objects, appreciate other people's tactile sensations, and detect risks such as hot or sharp objects. Tactile feeling is essential to many of our daily actions, yet with age, one's sensitivity to touch and capacity to perceive pain deteriorate [14, 15]. A person's touch sensation can be damaged by chronic conditions such as diabetes mellitus, cardiovascular disorders, stroke, Parkinson's disease, and arthritis. Some older adults have trouble distinguishing textures and objects just by touching them and it is possible that their response to touch will be delayed. Another complication is that with ageing, the pain threshold rises. Pain sensitivity can be heightened by certain medical conditions and drugs. Serious burns and wounds are more likely to occur in older adults before they experience any discomfort [16, 17].

One of the most inventive approaches to handling these types of health-care difficulties for the elderly is to ensure that future nurses have the necessary skills and self-confidence to nurse people with sensory impairments. As a result, nursing education needs to address issues within the health and social sectors pertaining to the ageing population. As a result, nursing educators must be creative when providing clinically relevant nursing education for knowing and qualified caregivers, incorporating not only social, constructive, and empirical components of learning, but also student learning [18, 19]. Nursing students who live with elderly people should have a priority of getting educational sessions on sensory impairments to know how to handle and communicate with elderly people living with sensory impairments [20].

Both age-related sensory impairments and population demographic change are substantial health-care challenges so, nurses, health, and social care providers should have knowledge and confidence in caring for older adults with sensory impairments. However, many health care professionals, particularly gerontological nurses, have a poor understanding of dual sensory impairment effects. Because nurses will be assessing, planning, implementing, and evaluating care plans for older adults with sensory impairments on a regular basis, they need to have the information and skills requisite to provide informed and compassionate care [19, 21].

The understanding and practise of younger people regarding sensory impairments, as well as how to manage older persons with issues that result in sensory impairments, is an essential topic to research. As a result, this study included first-year nursing students from three Egyptian universities: South Valley, Assuit, and Tanta Universities. Therefore, this study aims to assess the effect of providing nursing guidelines education on first-year nursing students' knowledge and reported practice towards sensory impairment among the elderly.

### Research hypothesis

Nursing students’ knowledge and reported practice towards sensory impairment among the elderly will be improved after the implementation of the nursing guidelines.

## Methods

### Study design

A pre-and post-test research design was utilized in this study to assess first-year nursing students’ knowledge and reported practice.

### Study setting

The study was conducted in three Faculties of Nursing at South Valley, Assiut, and Tanta Universities. The researchers selected these three universities to represent nursing students from three different regions of Egypt (South, middle and North Egypt). The first author had selected three major universities from three different regions of the country. Then she contacted two representatives from these universities.

### Subjects

A purposive sample of the first-year nursing students in the three previous mentioned faculties of nursing.

#### Inclusion criteria

First-year nursing students who live in households with elderly people accepted to participate in the study. The researchers selected the first-year nursing students because they would not have been taught any topic related to the study. The number of first-year nursing students for the 2020/2021 academic year and those who live with elderly people is shown in Table 1.

**Table 1:** Number of first-year nursing students

University	Number of first-year nursing students for the 2020/2021 academic year	Number of first-year nursing students who live with elderly people
South Valley	370	120
Assiut	822	269
Tanta	480	142
**Total**	1672	531

The sample size was 531 students

#### Development of pre-and post-test

The pre-and post-test were developed by the researchers based on reviewing the current related literature [22–24]. The data collected was about their socio-demographic background, their knowledge of the senses and their implementation of this knowledge in practice. The tests were placed online and included the following three parts.

Part 1 Students’ socio-demographic data – such as age, gender, address and university name.

Part 2 Assessment of students’ knowledge about the five senses and changes in these senses among the elderly as the following five sections: -

- Sense of vision – includes seven questions related to changes in sense of sight and effects of impaired vision.
- Sense of hearing – includes six statements related to effects of impaired hearing on the elderly.
- Sense of taste and smell– includes five statements related to effect of impaired sense of taste and smell on the elderly.
- Sense of touch – includes four statements related to effect of impaired sense of touch on the elderly.

Part 3 Assessment of students’ reported practice for coping with changes in these senses among elderly as the following five sections.

- Sense of vision – includes 14 statements about how to cope with the changes that resulted from poor vision, four statements about communication skills with the elderly who suffer from poor eye vision, and six statements about securing the environment surrounding the elderly to prevent accidents.
- Sense of hearing – includes 17 statements about how to cope with the changes that resulted from poor hearing.
- Senses of taste and smell – includes 11 statements about how to cope with changes that result from impaired senses of taste and smell.
- Sense of touch – includes five statements about how to cope with changes that result from an impaired sense of touch.

#### Scoring system

A score of one was given for each correct answer and zero for an incorrect answer or if the answer was not known.

#### Validity and reliability

A pilot of the test was undertaken to ascertain its validity and reliability. The purpose of the pilot study was to ensure the clarity of items and their comprehension, applicability and relevance to the questions. It also aimed to identify obstacles and problems that may occur during data collection, test the wording of the questions and estimate the time required to collect the study sample. To test validity, five experts from the Community Health Nursing Department were invited to comment on the test and give their feedback. To calculate validity, each of the 69 questions was rated from 1 to 5 degrees. The validity equation is the sum total number of questions that were rated 3 to 5 degrees from each one of the Jury Committees (65 + 63 + 62 + 68 + 66 = 324) divided by the total number of the questions multiplied by the number of jury numbers (69 × 5 = 345). The test is valid at 0.94 (324 ÷ 345 = 0.94).

To estimate the reliability, Cronbach's Alpha test was calculated and resulted in 0.765 for the knowledge part and 0.833 for the reported practice part which indicates that the questionnaire is reliable. The pilot study was applied before beginning of data gathering with 103 (10%) of the total number of first-year nursing students who then were excluded from the study sample due to test modifications.

#### Methods of data collection

The study was conducted in four phases as follows:

##### Phase 1

Pre-intervention assessment (pre-test)– The data were collected by administering the piloted test online using Google Form. Each studied subject was given the link to the online test to collect the baseline data about their current knowledge and reported practice about sensory impairments.

##### Phase 2

Nursing guidelines development – The guidelines were developed based on the results from Phase 1 and a recent relevant literature review [25–27].

##### Phase 3

A nursing guidelines implementation (intervention) – Four nursing guidelines education sessions (one hour session per week) were conducted through a Zoom application for the students. These sessions included explanations of five sense age-related changes among the elderly, effects of these changes and how to manage these changes.

##### Phase 4

Post-test assessment – One month following the education sessions the same test was used to evaluate the effect of the nursing guidelines on students' knowledge and reported practices. The data collection started in January 2021 and was completed in August 2021.

### Statistical analysis

Sociodemographic data such as age groups, sex, and residence were calculated using SPSS (version 23) and reported as frequency and percentage. For quantitative data, means and standard deviations (x ± s) were used for description. The chi-square test was used to compare frequency of correct and incorrect answers about knowledge and reported practice questions between pre-and post-intervention. Paired samples t-test was used to compare the means of total score of knowledge and reported practice between pre-and post-intervention. ANOVA test was used to check correlation between sociodemographic data and total score of knowledge and reported practice questions between pre and post intervention. Pearson correlation coefficient test was used to compare score of knowledge and reported practice during pre and post intervention (pre- and post-test). *P* value < 0.05 was adopted to interpret the significance findings. A high significance was adopted at *P* value < 0.01.

## Results

### Sociodemographic data

This study included 441 first-year nursing students aged 18–19 years old and 90 first-year nursing students aged 20 or more. There were 343 females compared to 188 males. Majority of the participants (83.1%) were aged between 18–19 years with a mean age of 18.98 ± 0.87 (18.0–22.0) and 64.6% (*n* = 343) of them were female. In terms of university locations, 50.7% were from Assiut University, 26.7% from Tanta University, and 22.6% were from South Valley University, with majority of the participants 71.8% were from a rural area (Table 2).

**Table 2:** Socio-demographic characteristics of the participants

Socio-demographic characteristics	No. (531)	%
**Age** (years):		
**18–20**	441	**83.1%**
> 20	90	16.9%
Mean ± SD (Range)	**18.98 ± 0.87 (18.0–22.0)**	
**Sex:**		
Male	188	35.4%
**Female**	343	**64.6%**
**Residence:**		
Rural	381	**71.8%**
Urban	150	28.2%
**University:**		
Assiut	269	**50.7%**
South Valley	120	**22.6%**
Tanta	142	**26.7%**

### Sense of vision

The percentage of students who correctly answered knowledge questions for a sense of vision among elderly people improved after receiving the educational nursing guidelines (post-test) compared to the pre-test. There was a highly statistically significant difference for most knowledge statements (*P* ≥ 0.05) except for two statements ‘the elderly person’s ability to focus decreases to see one thing at different distances’, and ‘vision impairment will affect the increased incidence of falls’ (*P* = 0.220) (Table 3). Furthermore, the percentage of students who correctly answered the questions related to reported practice (adaptation methods, communication skills, and methods of securing the environment) improved in the post-test compared to the pre-test. A highly statistically significant difference was calculated for all statements (*P* > 0.05) (Tables 4 and 5).

**Table 3:** Participants’ knowledge toward sense of vision in the pre and post intervention

**Knowledge toward sense of vision**	**Pre intervention** *n* = 531				**Post intervention** *n* = 531				***P*-value**
	**Correct**		**Incorrect**		**Correct**		**Incorrect**		
	**No**	**%**	**No**	**%**	**No**	**%**	**No**	**%**	
The sense of vision decreases in the elderly	510	96.0	21	4.0	531	100.0	0	0.0	0.000*
Does the elderly ability to see clearly impaired	514	96.8	17	3.2	527	99.2	4	0.8	0.004*
Does the elderly person’s ability to focus decreases to see one thing at different distances (especially seeing small nearby objects such as the sewing needle hole- the warnings written on the medicine box. Also, reading the small font	498	93.8	33	6.2	507	95.5	24	4.5	0.220
Does the elderly person’s ability to see small things close to him, such as the hole of a sewing needle, and to read the warnings written on the medicine box in small font, decrease?	444	83.6	87	16.4	487	91.7	44	8.3	0.000*
Does the elderly person can differentiate between different colours (especially blue- green- violet)?	186	35.0	345	65.0	246	46.3	285	53.7	0.000*
Does the elderly person’s ability to see in dim light and adapt when moving from light to dark and vice versa decreases?	430	81.0	101	19.0	464	87.4	67	12.6	0.004*
Does the elderly's ability to see and adapt to bright light increase?	222	41.8	309	58.2	257	48.4	274	51.6	0.031*
Does the elderly's ability decrease to see distances correctly?	433	81.5	98	18.5	483	91.0	48	9.0	0.000*
Does impair vision affect daily activities such as: walking- going outside- moving to/from the bed or chair?	377	71.0	154	29.0	465	87.6	66	12.4	0.000*
Does impair vision affect the increased incidence of falls (falls)?	498	93.8	33	6.2	507	95.5	24	4.5	0.220

^*^ Statistically Significant Differences, Chi-square test was used

**Table 4:** Participants’ adaptation methods to vision impairment among the elderly in the pre and post intervention

**Methods used to adapt vision impairment**	**Pre-test** *n* = 531				**Post-test** *n* = 531				***P*-value**
	**Correct**		**Incorrect**		**Correct**		**Incorrect**		
	**No**	**%**	**No**	**%**	**No**	**%**	**No**	**%**	
Using vision aids such as glasses, magnifying lenses or electronic devices	473	89.1	58	10.9	516	97.2	15	2.8	0.001*
Using the colours that the elderly see well (yellow- orange- red) in painting walls, doors and stairs, as well as using cups and dishes of the same colours	300	56.5	231	43.5	448	84.4	83	15.6	0.001*
Printing on white backgrounds and using black and white for books and brochures that the elderly read	424	79.8	107	20.2	459	86.4	72	13.6	0.004*
When printing books or the result of a history wall, or newspapers for the elderly, the font size must not be less than 14	427	80.4	104	19.6	460	86.6	71	13.4	0.006*
When writing any printed or other instructions for the elderly, the Roman or Serif-style type must be used	262	49.3	269	50.7	394	74.2	137	25.8	0.001*
Encouraging the elderly to go for an eye examination every year unless he suffers from problems that require immediate examination	447	84.2	84	15.8	488	91.9	43	8.1	0.001*
Covering the windows with curtains to prevent the light from shining	293	55.2	238	44.8	415	78.2	116	21.8	0.001*
Using bright lighting to see things	135	25.4	396	74.6	178	33.5	353	66.5	0.004*
Avoid using shiny surfaces and utensils	303	57.1	228	42.9	431	81.2	100	18.8	0.001*
Keeping a light at night, especially in bathrooms and bedrooms	453	85.3	78	14.7	494	93.0	37	7.0	0.001*
Using usual (yellow) bulbs are better than fluorescent (neon) bulbs	164	30.9	367	69.1	317	59.7	214	40.3	0.001*
Giving the elderly enough time and opportunity to adapt to seeing things when moving from light to dark and vice versa	491	92.5	40	7.5	518	97.6	13	2.4	0.001*

^*^ Statistically Significant Differences, Chi-square test was used

**Table 5:** Participants’ communication skills and the environment securing methods in the pre and post intervention

**Communication skills used to adapt vision impairment**	**Pre-test** *n* = 531				**Post-test** *n* = 531				***P*-value**
	**Correct**		**Incorrect**		**Correct**		**Incorrect**		
	**No**	**%**	**No**	**%**	**No**	**%**	**No**	**%**	
**Communication skills with the elderly who has poor vision:**									
Introducing oneself when entering an elderly person every time	474	89.3	57	10.7	498	93.8	33	6.2	0.008*
Telling the elderly person in detail what you are going to do	466	87.8	65	12.2	505	95.1	26	4.9	0.000*
Communicating with the elderly by relying on other senses such as hearing and touch- with the possibility of telling him about this	463	87.2	68	12.8	494	93.0	37	7.0	0.001*
If the elderly person is blind, he must be told when he finishes talking and leaves the place	450	84.7	81	15.3	468	88.1	63	11.9	0.107
**Methods of securing the environment around the elderly to prevent accidents practice:**									
Arranging and clearing floors and stairs from any obstacles such as carpets, furniture (with sharp edges), wires and electrical connections	505	95.1	26	4.9	520	97.9	11	2.1	0.012*
The fewer things in the environment surrounding the elderly, the greater the safety	489	92.1	42	7.9	510	96.0	21	4.0	0.006*
Fixing the places of things surrounding the elderly	460	86.6	71	13.4	487	91.7	44	8.3	0.008*
Awareness of the elderly about changes in the places surrounding him	473	89.1	58	10.9	506	95.3	25	4.7	0.000*
Serving food in one fixed place	147	27.7	384	72.3	178	33.5	353	66.5	0.039*
Informing the elderly when moving and being in a new place by introducing him to the rooms, places of things and also people	470	88.5	61	11.5	506	95.3	25	4.7	0.000*
Offering to help the elderly and ask him how he can be helped without insisting on my opinion	458	86.3	73	13.7	487	91.7	44	8.3	0.004*

^*^ Statistically Significant Differences, Chi-square test was used

### Sense of hearing

The percentage of students who correctly answered knowledge questions improved in the post-test compared to the pre-test. There is a highly statistically significant difference between the studied subject as regards almost every statement of knowledge in sense of hearing (*P* = 0.001) except in one statement (‘I hear you, but I cannot distinguish what you are saying’) (*P* = 0.065). The percentage of students who correctly answers reported practice questions improved in the post-test compared to the pre-test as regards the methods they used to adapt hearing impairment among the elderly. There is a highly statistically significant difference regarding most statements of methods they used to adapt hearing impairment among the elderly (*P* = 0.001) except in statements about ‘ensuring the safety of hearing aids’, ‘Preparing environment by reducing noise elements such as: turning off the TV, closing the windows’, and ‘Using advanced devices such as a bell with light’ (*P* = 0.067, 0.107, 0.091 respectively) (Tables 6 and 7).

**Table 6:** Participants’ knowledge toward sense of hearing among elderly people in the pre and post intervention

**Knowledge about sense of hearing**	**Pre-test** *n* = 531				**Post-test** *n* = 531				***P*-value**
	**Correct**		**Incorrect**		**Correct**		**Incorrect**		
	**No**	**%**	**No**	**%**	**No**	**%**	**No**	**%**	
Sense of hearing decrease in the elderly?	464	87.4	67	12.6	496	93.4	35	6.6	0.001*
**Signs of impaired hearing in the elderly**									
The elderly people ask more than once	434	81.7	97	18.3	486	91.5	45	8.5	0.001*
Statement (I hear you, but I cannot distinguish what you are saying.)	268	50.5	263	49.5	298	56.1	233	43.9	0.065
See the speaker is mumbling	244	46.0	287	54.0	301	56.7	230	43.3	0.001*
No loud sounds are heard	81	15.3	450	84.7	166	31.3	365	68.7	0.001*
Understand speech incorrectly and does not follow the context of the conversation	204	38.4	327	61.6	264	49.7	267	50.3	0.001*
**Effect of poor hearing on the elderly**									
Isolation and withdrawal from various social events	468	88.1	63	11.9	517	97.4	14	2.6	0.001*
Frustration and depression	276	52.0	255	48.0	313	58.9	218	41.1	0.022*

^*^ Statistically Significant Differences, Chi-square test was used

**Table 7:** Participants’ adaptation methods to hearing impairment among elderly people in the pre and post intervention

**Methods to adapt hearing impairment**	**Pre-test** *n* = 531				**Post-test** *n* = 531				***P*-value**
	**Correct**		**Incorrect**		**Correct**		**Incorrect**		
	**No**	**%**	**No**	**%**	**No**	**%**	**No**	**%**	
Periodic check-up and visit to an ear, nose and throat doctor	501	94.4	30	5.6	515	97.0	16	3.0	0.035*
Ensuring the safety of hearing aids	498	93.8	33	6.2	511	96.2	20	3.8	0.067
Speaking in a clear and audible voice	402	75.7	129	24.3	509	95.9	22	4.1	0.001*
Sitting in front of elderly person face to face during talking	484	91.1	47	8.9	505	95.1	26	4.9	0.011*
Attracting the elderly person's attention before he starts talking	491	92.5	40	7.5	515	97.0	16	3.0	0.001*
Preparing environment by reducing noise elements such as: turning off the TV, closing the windows	476	89.6	55	10.4	491	92.5	40	7.5	0.107
Using facial and body expressions, and resorting to writing if the elderly person is educated	443	83.4	88	16.6	497	93.6	34	6.4	0.001*
Asking elderly person short and simple phrases, giving enough time to understand and respond	444	83.6	87	16.4	504	94.9	27	5.1	0.001*
Talking out loud	196	36.9	335	63.1	292	55.0	239	45.0	0.001*
Using more than one word or phrase according to the level of education and living of the elderly, taking into account the use of appropriate phrases	408	76.8	123	23.2	460	86.6	71	13.4	0.001*
Covering the mouth while talking or eating and chewing gum	265	49.9	266	50.1	411	77.4	120	22.6	0.000*
Talking near the elderly person's ear	393	74.0	138	26.0	466	87.8	65	12.2	0.001*
Not to speak in the direction of the ear that hears the most	212	39.9	319	60.1	369	69.5	162	30.5	0.000*
Using advanced devices such as a bell with light	438	82.5	93	17.5	458	86.3	73	13.7	0.091

^*^ Statistically Significant Differences, Chi-square test was used

### Sense of taste and smell

The percentage of students who correctly answered knowledge and reported practice questions improved in the post-test compared to the pre-test. A highly statistically significant difference was calculated for all statements (*P* > 0.05) (Table 8).

**Table 8:** Participants' knowledge and reported practice towards sense of taste and smell in the pre and post intervention

**Knowledge**	**Pre-test** *n* = 531				**Post-test** *n* = 531				***P*-value**
	**Correct**		**Incorrect**		**Correct**		**Incorrect**		
	**No**	**%**	**No**	**%**	**No**	**%**	**No**	**%**	
Do the senses of taste and smell impaired in the elderly?	349	65.7	182	34.3	481	90.6	50	9.4	0.000*
What are the factors that weaken (affect) senses of taste and smell other than age?									
• Some diseases such as diabetes and cancer	285	53.7	246	46.3	326	61.4	205	38.6	0.011*
• Smoking	302	56.9	229	43.1	383	72.1	148	27.9	0.000*
• Poor oral hygiene and dentures	323	60.8	208	39.2	366	68.9	165	31.1	0.006*
What are the signs of impaired sense of taste and smell in the elderly?									
• Poor ability of the elderly to distinguish the smell of food	109	20.5	422	79.5	140	26.4	391	73.6	0.025*
• Weak ability of the elderly to judge the taste of food	416	78.3	115	21.7	458	86.3	73	13.7	0.001*
• Consuming more salt or sugar than usual	318	59.9	213	40.1	381	71.8	150	28.2	0.000*
What are the effects of poor sense of taste and smell on the elderly?									
• Causing many fire accidents	177	33.3	354	66.7	226	42.6	305	57.4	0.002*
• Malnutrition	356	67.0	175	33.0	403	75.9	128	24.1	0.001*
• Food poisoning	250	47.1	281	52.9	303	57.1	228	42.9	0.001*
• High blood pressure or blood sugar	297	55.9	234	44.1	353	66.5	178	33.5	0.000*
**Reported practice**									
Presenting food in an attractive way	393	74.0	138	26.0	495	93.2	36	6.8	0.000*
Cutting food into large pieces is better than using a blender	107	20.2	424	79.8	297	55.9	234	44.1	0.000*
Eating in a group or with the rest of the family	399	75.1	132	24.9	447	84.2	84	15.8	0.000*
Use lemon, vanilla, cinnamon and spices instead of salt	361	68.0	170	32.0	465	87.6	66	12.4	0.000*
Paying attention to oral and dental hygiene or dentures	418	78.7	113	21.3	480	90.4	51	9.6	0.000*
Diversity of nutrients in one meal	398	75.0	133	25.0	479	90.2	52	9.8	0.000*
Write the date of preservation in the refrigerator on the food and review it before use	387	72.9	144	27.1	425	80.0	106	20.0	0.006*

^*^ Statistically Significant Differences, Chi-square test was used

### Sense of touch

The percentage of students who correctly answered knowledge and reported practice questions improved in the post-test compared to the pre-test. There is a highly statistically significant difference between the studied subjects as regards most statements of knowledge and reported practice related to the sense of touch (*P* = 0.001) except the statement of knowledge about ‘Delayed sensation of pain resulting from wounds, burns and others’ (*P* = 0.058) (Table 9).

**Table 9:** Participants' knowledge and reported practice towards sense of touch in the pre and post intervention

**Knowledge**	**Pre-test** *n* = 531				**Post-test** *n* = 531				***P*-value**
	**Correct**		**Incorrect**		**Correct**		**Incorrect**		
	**No**	**%**	**No**	**%**	**No**	**%**	**No**	**%**	
Decrease sense of touch and pain	380	71.6	151	28.4	452	85.1	79	14.9	0.001*
Delayed sensation of pain resulting from wounds, burns and others	342	64.4	189	35.6	371	69.9	160	30.1	**0.058**
Delayed reaction	425	80.0	106	20.0	468	88.1	63	11.9	0.001*
Poor knowledge of the sharpness of some tools, such as a knife or scissors	278	52.4	253	47.6	328	61.8	203	38.2	0.002*
**Reported Practice**									
Open hot water tap first, then cold water, and vice versa when closing	133	25.0	398	75.0	174	32.8	357	67.2	0.006*
Use a thermometer to measure the temperature of the water	442	83.2	89	16.8	483	91.0	48	9.0	0.001*
Lower the water temperature to 100 degrees Fahrenheit (37 degrees Celsius) as a medium	397	74.8	134	25.2	465	87.6	66	12.4	0.001*
Not to use sharp tools or furniture in the environment (kitchen, rooms)	418	78.7	113	21.3	486	91.5	45	8.5	0.001*
Noting the elderly to report when they feel any pain, even if it is slight	433	81.5	98	18.5	499	94.0	32	6.0	0.001*

^*^ Statistically Significant Differences

Chi-square test was used

### Overall knowledge and reported practice scores of the five senses

**Fig. 1:**
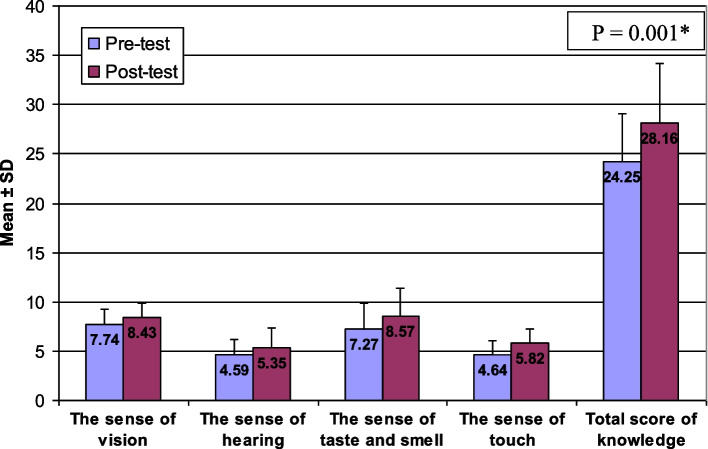
Participants’ knowledge mean score about the five senses in pre and post intervention. * Paired sample t-test was used

**Fig. 2:**
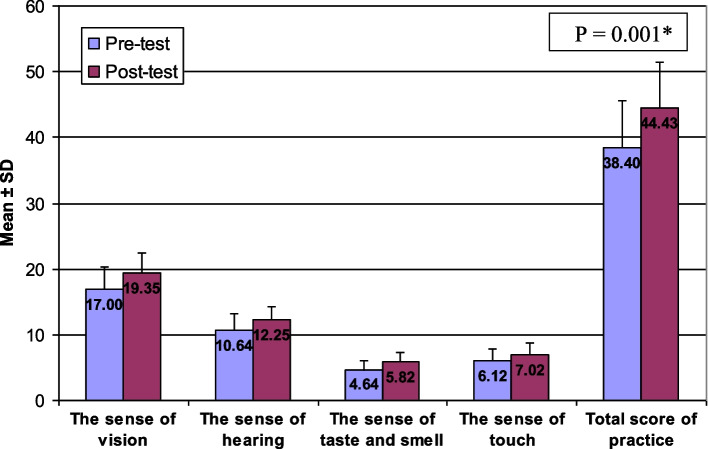
Participants’ reported practice means score about the five senses in the pre and post intervention. * Paired sample t-test was used

**Fig. 3:**
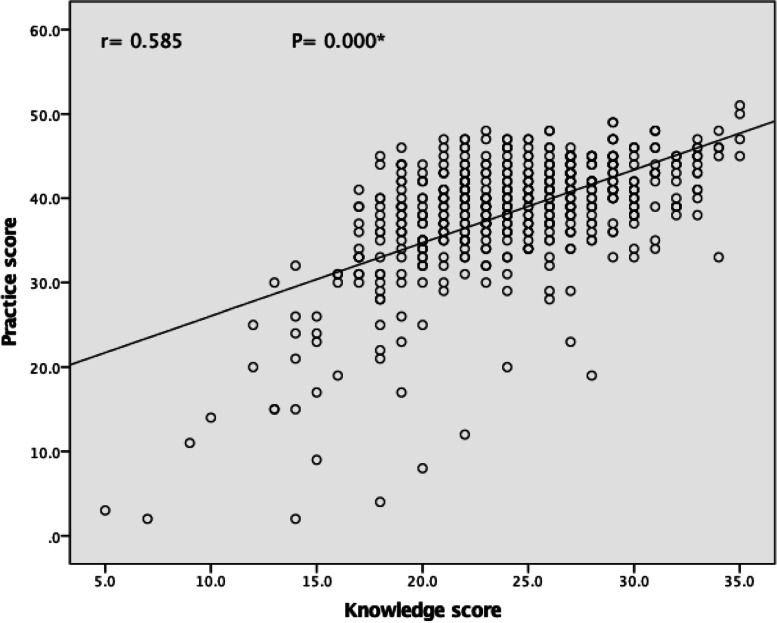
Correlation between participants’ knowledge and reported practice score about the five senses in the pre-intervention. *Pearson correlation was used

**Fig. 4:**
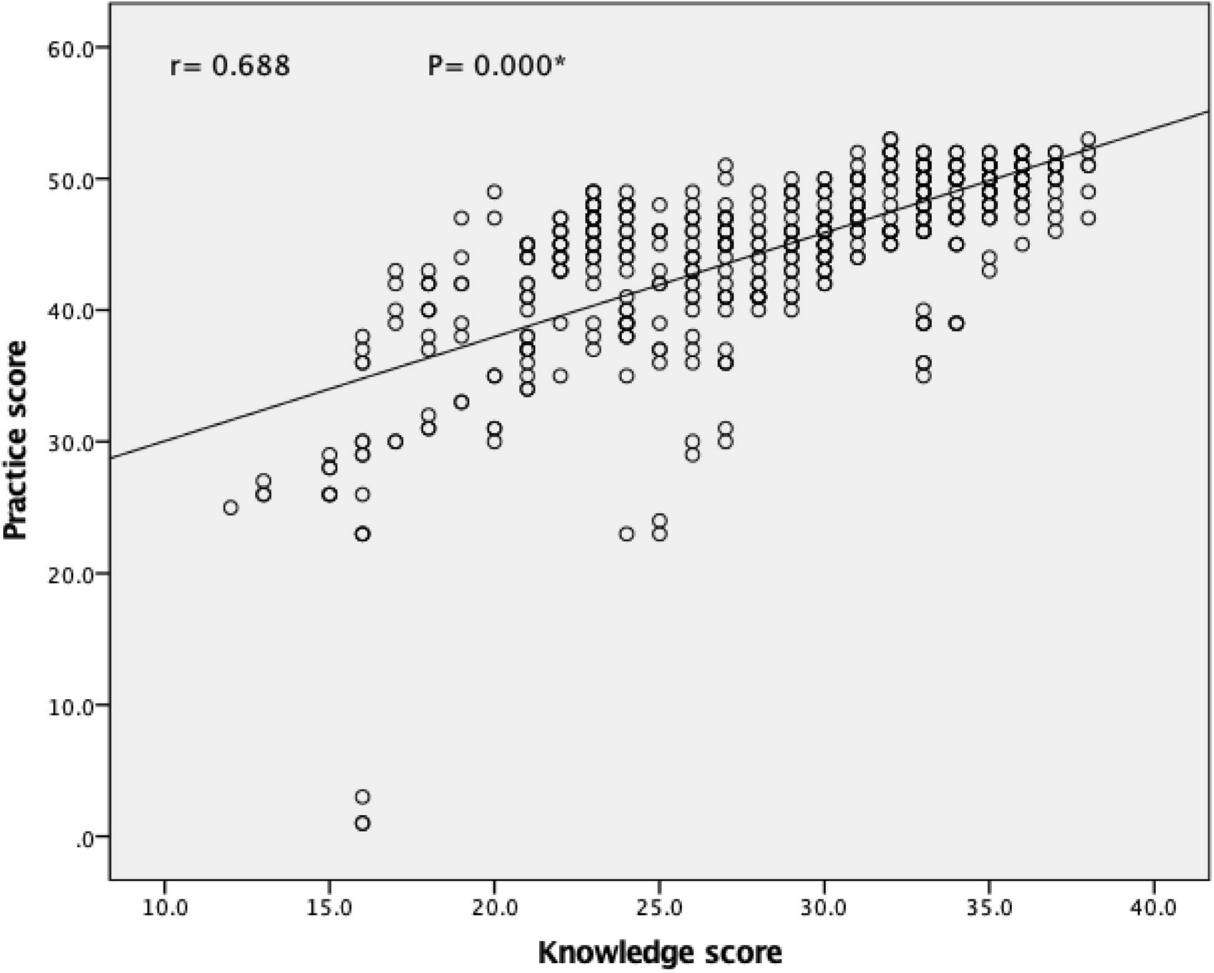
Correlation between participants’ knowledge and reported practice about the five senses score in the post intervention. *Pearson correlation was used

The mean score of the knowledge and reported practice for all five senses was increased in the post intervention compared to the pre intervention. At the baseline data, the total mean score of the students’ knowledge was 24.25, compared to 28.16 post intervention (Fig. 1). While the total mean score of students’ reported practice increased from 38.40 during pre-intervention to 44.43 post intervention (Fig. 2). Furthermore, Pearson correlation test showed a positive relationship between knowledge score and reported practice score at pre- and post intervention (*r* = 0.585, 0.688 respectively) (Figs. 3 and 4). Finally, there are no statistical significant differences between the students’ knowledge score, reported practice score and their personal data during pre and post intervention (*P* > 0.05) (Table 10).

**Table 10:** Comparing of knowledge and reported practice mean score of participants’ socio-demographic characteristics between pre and post intervention

Socio-demographic characteristics	Knowledge (maximum score 35)		Practice (maximum score 49)	
	**Pre-test** *n* = 531	**Post-test** *n* = 531	**Pre-test** *n* = 531	**Post-test** *n* = 531
	**Mean ± SD**	**Mean ± SD**	**Mean ± SD**	**Mean ± SD**
**Age: (years)**				
18 < 20	24.24 ± 4.79	28.05 ± 6.01	38.41 ± 6.67	44.40 ± 6.84
20 or more	24.28 ± 4.90	28.69 ± 6.08	38.32 ± 9.04	44.60 ± 7.45
*P*-value*	0.953	0.360	0.913	0.803
**Sex:**				
Male	24.14 ± 4.73	28.29 ± 6.13	38.53 ± 6.58	44.30 ± 7.38
Female	24.31 ± 4.85	28.08 ± 5.97	38.33 ± 7.40	44.50 ± 6.70
*P*-value*	0.691	0.704	0.757	0.750
**Residence:**				
Rural	24.35 ± 4.84	28.25 ± 5.80	38.28 ± 7.02	44.62 ± 6.34
Urban	23.99 ± 4.73	27.92 ± 6.57	38.70 ± 7.37	43.97 ± 8.27
*P*-value*	0.439	0.568	0.539	0.332
**University:**				
Assiut	24.05 ± 4.83	28.13 ± 5.99	38.95 ± 7.74	44.23 ± 7.53
South Valley	23.82 ± 4.42	28.38 ± 6.12	38.28 ± 6.75	44.74 ± 6.34
Tanta	24.99 ± 5.02	28.03 ± 6.04	37.44 ± 6.03	44.56 ± 6.26
*P*-value**	0.089	0.887	0.122	0.770

^*^ Independent samples t-test, ** ANOVA test

## Discussion

The aim of this study was to assess effect of teaching nursing guidelines related to sensory impairments on first-year nursing students' knowledge and reported practice towards sensory impairment among the elderly. The study results suggest that nursing guidelines sessions improve the first-year nursing students’ knowledge about sensory impairment among the elderly people. This improvement had a positive impact on the students’ reported practice. These results in the same line with the results of the study done by Macaden et al. who confirmed that first-year nursing students who participated in sensory simulation activity understanded what may be required of them when they communicate, deal with elderly people with sensory impairments [23]. The current findings show a highly statistically significant difference in knowledge scores for hearing, vision, taste, smell and touch deficits before and after the nursing guidelines sessions. These findings are similar to those of Walters et al. (2021), who investigated the effects of fidelity simulation on sensory impairments in order to create the necessary information and understanding to support persons with these impairments. The simulation was well accepted and had a favourable influence on students' knowledge, awareness and skills in treating patients with sensory impairments [28].

The results of the current study revealed that after implementing the study programme, first-year nursing students' knowledge about the five senses improved, and they received a high score on the reported practice regarding how to adapt to sensory impairment among older adults. This finding is consistent with Chen et al.’s (2015) findings from a study conducted at a nursing school in the Midwest of the US which found that students may be unaware of older individuals' feelings and experiences prior to experiencing aging-related changes themselves. Simulation activities, such as the Geriatric Medication Game, can be an effective way to teach empathy and compassion to students [29]. In this study, the researchers encouraged participant students to attend the sessions and to ask and discuss any questions they had about the session's content. In addition, the participant students should emphasise their complete understanding and maximize feedback. These prior reasons contributed to increase the students’ response rate.

According to the findings of this study, there is a highly statistically significant difference between the participants reported practice at pre-test and post-test level related to the methods they used to adapt hearing and vision impairment among elderly. This research is supported by the findings of Smith et al. (2018), who reported that only a small percentage of workshop participants felt secure in providing a full account of hearing requirements in their patients' treatment plans (*n* = 3, 7.3% and *n* = 3, 8.1%, respectively). Although confidence increased after the workshop, the numbers remained low (*n* = 16, 44.4% and *n* = 14, 41.4%, respectively) [30]. The students' reported practice score increased from pre-test to post-test. This could be explained by students learning more about sensory impairments and how to manage older adults with sensory impairments, as evidenced by a positive link between knowledge and reported practice. These findings are consistent with those of Smith et al. (2018), who found that providing accessible sensory impairment education to health and social care providers can improve care delivery to older adults [30].

## Conclusion

Based on the results of the current research, it was noticed that the nursing guidelines were effective in improving the mean score of knowledge and mean score of reported practice for the participants toward sensory impairment among elderly people. Before providing the intervention guidelines, the majority of the students reported low level of knowledge score and reported practice scores. After the application of the nursing guidelines the mean score of knowledge and reported practice of the studied sample improved. The study recommended that these nursing guidelines could be embedded within the undergraduate curriculum. Raising students’ awareness through providing lectures, and workshops on sensory impairment among elderly and how to deal with them, and train students on how to communicate with sensory impairment among elderly.

### Study strengths and limitations

This study has its strength. The new developed and validated tool was utilized to assess students’ knowledge and reported practice about the five senses among elderly people. However, the study has a few limitations; there was no control group to compare groups’ differences and the self-reported practice may be caused biased. The ideal assessment of practice is to observe the student when they communicate with elderly people having sensory impairments, therefore, further studies are required to measure the observed practice of nursing students.

## Data Availability

The datasets used and analyzed during the current study are available from the corresponding author on reasonable request.
